# A consensus-hemagglutinin-based vaccine delivered by an attenuated *Salmonella* mutant protects chickens against heterologous H7N1 influenza virus

**DOI:** 10.18632/oncotarget.16353

**Published:** 2017-03-18

**Authors:** Kim Je Hyoung, Irshad Ahmed Hajam, John Hwa Lee

**Affiliations:** ^1^ College of Veterinary Medicine, Chonbuk National University, Iksan Campus, Iksan 54596, Republic of Korea

**Keywords:** cell mediated immunity, hemagglutinin, humoral immunity, influenza, Salmonella Typhimurium

## Abstract

H7N3 and H7N7 are highly pathogenic avian influenza (HPAI) viruses and have posed a great threat not only for the poultry industry but for the human health as well. H7N9, a low pathogenic avian influenza (LPAI) virus, is also highly pathogenic to humans, and there is a great concern that these H7 subtypes would acquire the ability to spread efficiently between humans, thereby becoming a pandemic threat. A vaccine candidate covering all the three subtypes must, therefore, be an integral part of any pandemic preparedness plan. To address this need, we constructed a consensus hemagglutinin (HA) sequence of H7N3, H7N7, and H7N9 based on the data available in the NCBI in early 2012-2015. This artificial sequence was then optimized for protein expression before being transformed into an attenuated auxotrophic mutant of *Salmonella* Typhimurium, JOL1863 strain. Immunizing chickens with JOL1863, delivered intramuscularly, nasally or orally, elicited efficient humoral and cell mediated immune responses, independently of the route of vaccination. Our results also showed that JOL1863 deliver efficient maturation signals to chicken monocyte derived dendritic cells (MoDCs) which were characterized by upregulation of costimulatory molecules and higher cytokine induction. Moreover, immunization with JOL1863 in chickens conferred a significant protection against the heterologous LPAI H7N1 virus challenge as indicated by reduced viral sheddings in the cloacal swabs. We conclude that this vaccine, based on a consensus HA, could induce broader spectrum of protection against divergent H7 influenza viruses and thus warrants further study.

## INTRODUCTION

Avian influenza viruses (AIV) are important pathogens that cause huge economical losses to the poultry industry every year [1]. They are designated as highly pathogenic avian influenza (HPAI) or low-pathogenicity avian influenza (LPAI) based on the pathogenicity and virulence in chickens [2]. The HPAI and LPAI viruses cause acute systemic disease with high flock mortality and mild respiratory disease, respectively [2]. Outbreaks of HPAI and LPAI viruses belonging to H7 subtype in chickens have been reported in the past [3–5]. Infections with H7N3, H7N7, and H7N9 have not only infected poultry but also humans, which exhibited conjunctivitis or respiratory symptoms with limited human-to-human transmission [6–10]. There is a great concern that these H7 subtypes would acquire the ability to spread efficiently between humans, thereby becoming a pandemic threat [10]. Moreover, infections caused by these viruses are a major public health concern as it is unlikely that there will be pre-existing immunity against these subtypes in the human population. Therefore, a vaccine covering all the three subtypes must, therefore, be an integral part of any pandemic preparedness plan. Vaccination remains the most effective method to protect the population, including both humans and animals, against influenza infections [11, 12]. Currently available influenza vaccines can be divided into inactivated and live attenuated vaccines [12]. Although effective in controlling viral infections, these vaccination strategies require a large supply of specific-pathogen free (SPF) embryonated eggs and a long timeline that could be threatened during an influenza pandemic affecting both humans and poultry [11]. Moreover, these vaccines can mainly elicit neutralizing antibodies against the homologous virus but with limited activity against divergent viruses [13, 14], which is not an economically viable option for the poultry production. Therefore, several novel approaches avoiding the use of SPF eggs have been investigated to induce protective immunity against the key structural proteins of influenza viruses, for instance, hemagglutinin (HA). Some of these approaches include recombinant protein based vaccines [15], adenovirus based vaccines [16], and DNA plasmids [17, 18]. These promising vaccination strategies, especially DNA vaccines, allow for easier manipulation and faster production when compared to the conventional influenza vaccines. However, DNA vaccines are poorly immunogenic in nature and have thus shown insufficient protection against the virus infection [17, 18]. Therefore, an effective vaccination strategy should be devised which not only elicits potent immune responses but also allows easier manipulation, faster production, and protection against diverse strains of influenza viruses.

Influenza A viruses generally cause yearly epidemics and, potentially, pandemics when an influenza virus with a novel antigenically shifted HA emerges in a population resulting in widespread infection, high morbidity, and high mortality [19]. Influenza virus is made up of 11 proteins, and studies have shown that HA is no doubt the major target for generating protective immunity [11, 18]. Anti-HA antibodies have potential to block viral attachment and entry, and thus infection of the host cells subsequently [20]. The influenza viruses have the ability to continuously evolve either gradually through antigenic drift (point mutations) or rapidly through reassortment with another divergent virus (antigenic shift) [5]. Consequently, the immunity generated against one vaccine strain is only protective against another strain that shares antigenically related proteins. Therefore, influenza vaccines need to be annually updated pertaining to the antigenic changes of circulating field strains. Live bacterial vectors are a promising approach to deliver immunogenic proteins of AIV to the immune system [21]. Studies have demonstrated that attenuated *Salmonella* based system carrying various heterologous viral antigens elicit efficient systemic and mucosal immune responses [22]. In the present study, we exploited *Salmonella enterica* serovar Typhimurium (*S*. Typhimurium) system to deliver a consensus HA protein of H7N3, H7N7, and H7N9 in chicken model. This strategy is highly cost effective and allows for a quick response to novel influenza viruses, as it circumvents the need for a constant supply of eggs. Moreover, only antigenically important proteins from the influenza viruses are used to construct the vaccine, so the candidate offers the potential to differentiate vaccinated from infected animals.

Here we report the construction of an attenuated auxotrophic mutant of *S*. Typhimurium, JOL1863 strain, expressing and secreting a consensus HA based on available H7N3, H7N7, and H7N9 sequences. Our results show that JOL1863 immunization in chickens induced efficient humoral and cell mediated immune (CMI) responses, independently of the route of vaccination. We also show that JOL1863 drive efficient maturation of chicken monocyte derived dendritic cells (MoDCs), characterized by upregulation of costimulatory molecules and higher cytokine induction. Heterologous LPAI H7N1 virus challenge experiment further confirms that JOL1863 vaccination can protect chickens from viral shedding and may thus reduce the risk to transmit viruses to other species, and subsequent emergence of HPAI viruses affecting both humans and poultry. Taken together, these findings suggest that consensus HA based vaccine delivered by *Salmonella* represents one vaccine approach against influenza viruses and is thus worthy of further investigation.

## RESULTS

### Design of a prototype HA based vaccine delivered by an attenuated auxotrophic mutant of *Salmonella* Typhimurium

**Figure 1:**
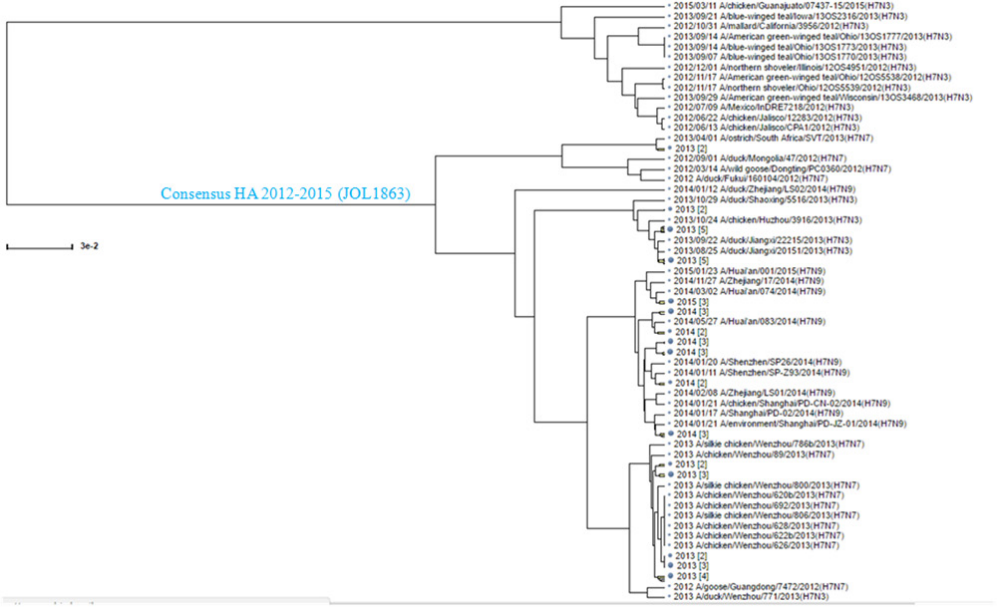
Phylogenetic analysis of the H7N1, H7N7, and H7N9 HA sequences The HA sequences of H7N1, H7N7, and H7N9 available in the Influenza Virus Resource data base in early 2012-2015 were aligned by Clustal W algorithm, and a consensus sequence was generated. The consensus sequence used in this study is shown in blue with the bacterial strain name indicated in parentheses.

The HA sequences of circulating H7N3, H7N7, and H7N9 influenza A viruses were aligned and a conserved consensus HA protein sequence was deduced from 49 sequences of H7N3, 64 of H7N7, and 282 of H7N9 viruses available in the GenBank in early 2012 to 2015 (Figure 1). To construct the *S*. Typhimurium mutant, strain JOL1863, expressing HA, the codon optimized synthetic HA consensus gene sequence was cloned into a constitutive expression vector, pMMP65, and subsequently propagated in *asd* mutated *Escherichia coli* as described previously [23]. The insertion of HA gene into pMMP65 vector was confirmed by digestion of positive clones with *EcoR1* and *HindIII* to release a fragment of 959 bp size. The resultant plasmid, pMMP65-HA, was electroporated into JOL912 strain for protein expression as described earlier [23], and the resultant clone was designated as JOL1863. Western blot analysis showed a protein band corresponding to 41 kDa, the expected size of our protein, and thus confirmed the expression of HA. The expressed protein was found both in supernatant and in periplasmic fractions of the bacterial cultures (Supplementary Figure 1). The expressed protein was found functionally active as it exhibited hemadsorption activity *in vitro*, as demonstrated by the chicken RBC agglutination.

### JOL1863 induces functional and protective antibody responses

**Figure 2:**
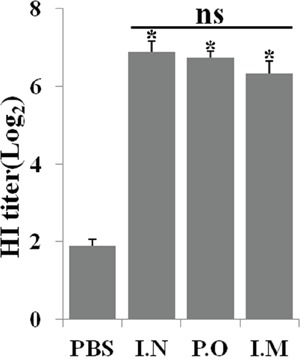
Specific antibody titers in chickens after immunization with JOL1863 Chickens were immunized with JOL1863 expressing HA, and serum samples were analysed for HI activity after 28 days post vaccination. Each data points represent mean ± S.D. of five chickens per group. p<0.05. ns, non-significant.

HI test reflects the level of functional antibodies that confers protective immunity against influenza infections [11]. To investigate the ability of JOL1863 to induce neutralizing antibodies, groups of chicken (n = 10) were vaccinated intramuscularly, nasally, or orally with 10^9^ JOL1863 CFU or 200 μl PBS, and blood was drawn on 28^th^ day post immunization for determination of HI titers. During our observation period of 6 weeks, we found that JOL1863 vaccination did not induce any symptoms of acute or subacute toxicity in chickens, thereby demonstrating that JOL1863 bacteria are well tolerated. Our results demonstrated that efficient neutralizing antibody responses were elicited in vaccinated chickens with respect to controls, independently of the route of vaccination. As shown in Figure 2, the HI titers were significantly higher (p<0.05) in immunized chickens as compared to the control group. However, no statistical significant difference was observed when neutralizing antibody titers were compared among groups which received JOL1863 vaccine.

### JOL1863 vaccination induces efficient systemic and mucosal antibody responses

**Figure 3:**
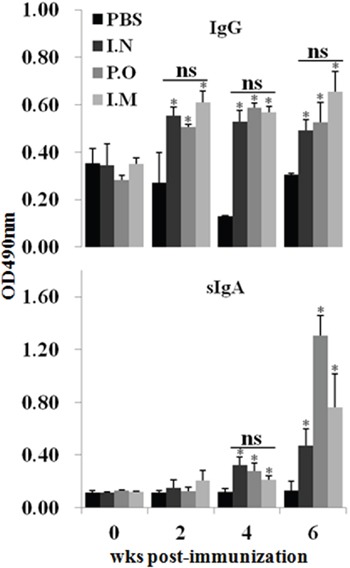
Effect of JOL1863 vaccination on the systemic IgG and mucosal IgA responses Immunization of chickens with JOL1863, delivered nasally (I.N), orally (P.O) or intramuscularly (I.M), improved both anti-IgG and anti-IgA antibody responses. The antibody IgG and sIgA levels were measured in serum and intestinal wash samples, respectively, at different time points post-vaccination by indirect ELISA. Each data points represent mean ± SD of five chickens per group. *p < 0.05. ns, non-significant.

To assess the effect of JOL1863 vaccination on systemic and mucosal antibody responses, indirect ELISA for IgG and IgA was performed on weekly collected post immunization sera and intestinal wash samples, respectively. The HA specific IgG and IgA responses were elicited independently of the route of immunization. The kinetics of IgG and IgA are shown in Figure 3.

JOL1863 vaccination induced significantly (P<0.05) higher HA specific systemic IgG responses as compared to the unimmunized control group (Figure 3). The IgG levels were detected at 14^th^ day post-vaccination, which maintained till 6^th^ wk in all the JOL1863 immunized groups. However, there was no statistically significant difference among JOL1863 immunized groups, suggesting that induction of IgG responses by *Salmonella* delivering HA is route independent. Our results further demostrated that JOL1863 vaccination was highly efficient in eliciting HA specific mucosal IgA responses. The IgA responses were significantly (p<0.05) higher in vaccinated chickens as compared to the control group. The IgA levels in all the immunized groups detected at 28^th^ day post immuniation and peaked at 6^th^ wk (Figure 3), irrespectively of the route of vaccination. Among vaccinated chickens, sIgA levels were found significantly (p<0.05) higher in oral group as compared to the nasal and intramuscular groups, which showed almost comparable levels.

### JOL1863 stimulate efficient maturation and activation of MoDCs

The elicitation of significant protective immune responses following JOL1863 vaccination suggests that the vaccine is efficiently taken up and processed by dendritic cells (DCs). Because DCs are potent APCs and are the only cells to prime naïve cells, we decided to evaluate the effect of JOL1863 and inactivated H7N1 virus on the maturation and activation of MoDCs. The MoDCs were stimulated for 48 hours with either JOL1863 (10 particles/cell) or inactivated H7N1 virus (500 ng/ml) or left unstimulated. Preincubation with JOL1863 resulted in an increased expression of MHC class II and CD40 molecules, which was significantly (p<0.05) higher as compared to the unstimulated cells (Supplementary Figure 2). Similar results were obtained using 500 ng/ml of H7N1 virus; however, the expression levels of CD40 were significantly (p<0.05) higher in JOL1863 stimulated cultures than in H7N1 stimulated cells.

### JOL1863 vaccination activate efficient CMI responses

**Figure 4:**
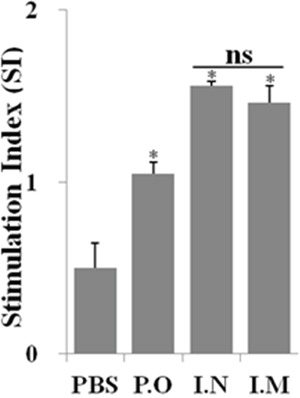
*In vitro* proliferations of lymphocytes from vaccinated chickens in response to recall HA antigen PBMCs from vaccinated chickens were restimulated with HA antigen (10 μg/ml) for 72 hours and lymphocyte proliferation was determined by MTT assay. Results are expressed as stimulation indices, defined as proliferation in response to recall antigen relative to the mock stimulated cells. Each data points represent mean ± SD of five chickens per group.*p < 0.05. ns, non-significant.

To assess the efficacy of the cellular immune responses stimulated by JOL1863 vaccination, the proliferative capacity of PBMCs after *in vitro* restimulation in the presence of HA protein was evaluated. Efficient proliferative responses were only observed in immunized chickens irrespective of the route of vaccination (Figure 4). Although the observed differences were not statistically significant (*p* >0.05), slightly better stimulation indexes were detected in animals receiving JOL1863 vaccination through intranasal route.

**Figure 5:**
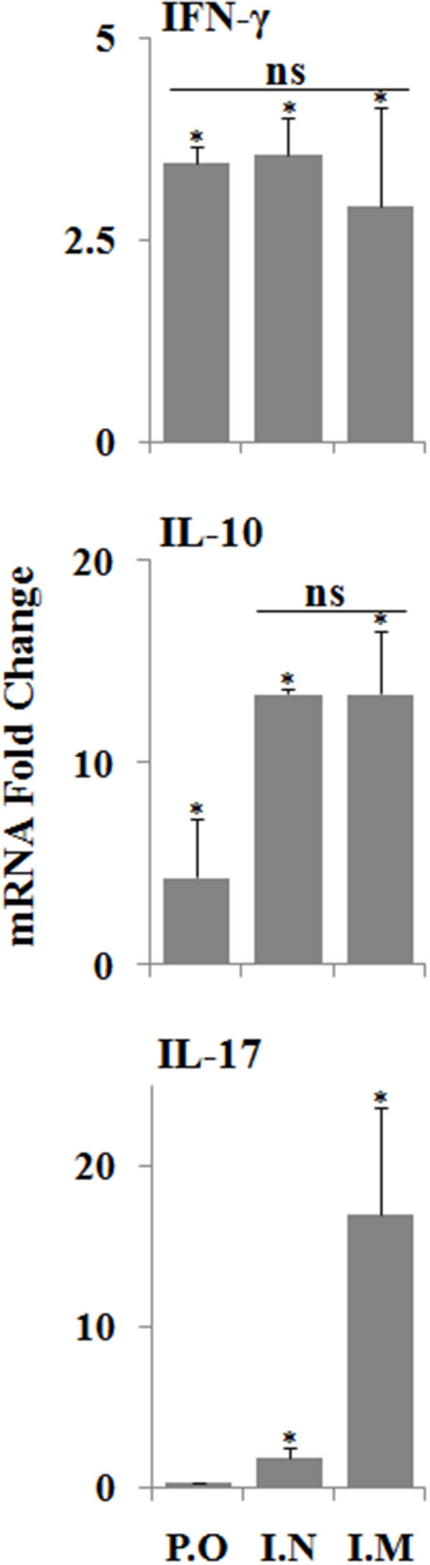
qRT-PCR analysis of cytokine gene expressions in PBMCs after stimulation with HA protein PBMCs collected from immunized chickens after 21 days post immunization were stimulated with HA antigen for 48 hours and analysed for induction of IFN-ϒ, IL-17 and IL-10 mRNA transcription levels by qRT-PCR assay. Results are expressed as fold change in mRNA transcription after HA protein stimulation of immunized groups compared to the HA treated naïve PBMCs. Gene expressions were normalized to GAPDH and mRNA levels of naive stimulated cells were used as calibrator. Data presented are mean ± SD of five chickens per group. *p < 0.05, ns, non-significant.

To complement the study of the cellular immune responses stimulated by JOL1863, we evaluated the capacity of PBMCs to produce IFN-**γ**, IL-17 and IL-10 cytokines in response to restimulation with HA protein. The mRNA expression levels of these cytokines were significantly (p<0.05) higher in immunized chickens as compared to the naïve chickens (Figure 5). The mRNA inductions of IFN-**γ** and IL-17 were increased by 2.9 to 3.5 fold and 0.5 to 17 fold, respectively, in comparison to the stimulated naive PBMCs. Though immunized groups did not show any significant differences in IFN-**γ** levels, however, IL-17 levels were significantly (p<0.05) higher in intramuscular group as compared to the nasal and oral groups. The IL-10 mRNA levels were increased by 4 to 13 folds compared to the stimulated naïve PBMCs. Among immunized groups, oral group has shown significantly (p<0.05) lower IL-10 levels while intramuscular and nasal groups showed almost comparable levels. Our results, thus, show that pre-incubation of PBMCs with JOL1863 results in efficient recall cellular activation and maturation as indicated by cytokine inductions, thereby explaining the improved immune responses observed using JOL912 as a delivery system for HA vaccine.

### JOL1863 vaccination protects chickens against lethal H7N1 challenge

The benchmark of an influenza vaccine is protection against a lethal virus challenge. Therefore to evaluate the efficacy of JOL1863 vaccination, all vaccinated and control chickens were challenged with 10^6^EID50 of heterologous H7N1 virus. Subsequently, cloacal wash samples were collected on 1^st^, 3^rd^, 5^th^ and 7^th^ day post challenge for determination of viral load by qRT-PCR assay. Chickens vaccinated with JOL1863 showed significantly (p<0.05) lower viral load as compared to the unimmunized control chickens (Table 1). Within the immunized groups, orally vaccinated chickens showed viral load only on 3^rd^ day post-challenge while intramuscularly vaccinated chickens showed on 3 and 5^th^ day post challenge. In case of intranasally immunized chickens, the viral load was detected from day 1 to 7 post-challenge, however, it was significantly (p<0.05) lower compared to the control group (Table 1).

**Table 1:** Protective efficacy of the JOL1863 vaccination against heterologous LPAI H7N1 challenge

dpc	number ofbirds	CON	P.O	I.N	I.M
1 Day	12345	10458.19112300.9116438.710379.85-	-----	1021.68----	-----
3 Day	12345	110374.4101151.410488.71--	-----	10682.29--11195.63-	1021.02----
5 Day	12345	11861791004.1971010.764--	--1019.313--	10583.41----	1081.986----
7 Day	12345	1097.193----	-----	10989.76----	-----

Abbreviations: dpi, days post challenge; Con, P.O, I.N, and I.M represent vaccination groups.

The protective efficacy was determined by estimation of H7N1 viral copy numbers in the cloacal swab samples of the immunized chickens (n=5) after challenge with the lethal H7N1 virus.

To complement our protected studies, we evaluated the T cell count in immunized and control chickens after challenge with H7N1 virus. The immunized groups showed significantly (p<0.05) higher percentage of CD4+ T cells as compared to the control group (Supplementary Figure 3), suggesting that vaccinated chickens showed better HA specific recall protective responses. Consequently, immunized groups showed higher percentage of protection upon lethal viral challenge as compared to the naïve chickens.

## DISCUSSION

Our goal was to investigate whether an attenuated auxotrophic mutant of *S*. Typhimurium delivering a consensus HA vaccine can elicit a significant immune response and protection in chickens against a lethal challenge of heterologous H7N1 influenza virus. A major obstacle in vaccine development against viruses such as influenza is the extent of genetic diversity [24]. Usually neutralizing antibodies raised by one vaccine strain provides little or no protection against the heterologous strain. One approach to minimize the sequence diversity between a vaccine strain and circulating viruses is to create a consensus sequence to centralize the immunogenicity of the vaccine antigen. Thus, we generated a HA consensus sequence based on the data available in the NCBI, and subsequently the sequence was cloned and expressed in *S*. Typhimurium. A major concern of using this approach is that such a consensus sequence may not yield a structurally and functionally intact protein, and therefore it is important to evaluate the biological activity of the protein *in vitro*. Our results demonstrated that HA protein was biologically active as it was found to exhibit hemadsorption activity *in vitro*. Previously, attenuated *Salmonella* system has been used to deliver HA based DNA vaccines against highly pathogenic H5N1 influenza virus, and partial protection was conferred against the virus infection [17, 22]. A potential limitation with the DNA vaccines delivered through bacteria is the inability to stably maintain the plasmid DNA inside the bacteria. Moreover, the gene expression of heterologous antigens encoded by DNA is inefficient, resulting in the poor immunogenicity of the vaccine [17]. Thus, in the present study we constructed JOL1863, an attenuated auxotrophic mutant of *S*. Typhimurium expressing a consensus HA protein, and used as a vaccine candidate in chickens. *Salmonella* based HA vaccines have certain advantages over conventional influenza vaccines. These vaccines are readily amenable to modifications once the circulating strains are identified and can be rapidly produced in large quantities without the need of specific cell culture conditions, as is the case with whole virus based influenza vaccines. Moreover, the delivery of HA by JOL1863 provides an appropriate danger signals to the immune system, acting as natural adjuvant, thereby promoting efficient maturation and activation of DCs, which is prerequisite for the induction of potent adaptive immune responses [24, 25]. The presentation of antigens by DCs in the absence of proper costimulatory molecules may even promote tolerance, thereby leading to immune escape [25]. In the present study, we investigated the potential of JOL1863 to induce maturation and activation of chicken MoDCs. We noticed that JOL1863 delivers efficient maturation signals to MoDCs as evidenced by the increased expression of MHC-II and CD40 expression levels. Subunit vaccines are usually poorly immunogenic in nature, and therefore need an appropriate adjuvant to activate DCs for efficient antigen processing and presentation to naïve T cells [26]. Previous studies show immunization of mice with recombinant HA protein without any use of adjuvants elicits very low antibody responses, and consequently fails to protect the mice against lethal influenza virus challenge [27]. This suggests that subunit proteins are not presented properly to the immune system due to lack of efficient maturation signals, and therefore necessitating an appropriate adjuvant in the vaccine formulation. The increased expression of costimulatory molecules observed in the present study indicated that *Salmonella* delivers efficient maturation signals to APCs and therefore efficient presentation of HA antigen to naïve T cells. This could explain the increased immunogenicity of HA observed in the current study.

Induction of HA-specific neutralizing antibody responses in peripheral blood circulation strongly correlates with the recovery from the clinical disease and protection from subsequent challenge infection [11]. The present study demonstrates that a consensus HA based vaccine induced elevated levels of anti-HA neutralizing antibodies and subsequent protection against H7N1 challenge. The neutralizing antibody induction was independent of the route of vaccination, and consistently the protection against infection was also independent of the route of immunization. Sequence analyses revealved that H7N1 differs from H7N3, H7N7, and H7N9, indicating that JOL1863 has the potential to provide a broad spectrum of protection against divergent strains of H7 viruses. Our results are in accord to the previous reports, which show that consensus HA based vaccines can provide protection against divergent clades of H5N1 influenza viruses [11, 17]. This suggests that consensus HA based vaccine represents a promising approach to protect human and animal population against divergent strains of influenza viruses.

As influenza viruses are transmitted mainly at mucosal sites, induction of a potent mucosal immunity is a critical parameter in evaluating a good vaccine candidate. The induction of IgA response at mucosal surfaces has the ability to prevent viral attachment to epithelial cells and infection of the host cells subsequently [28, 29]. In the present study, we found that JOL1863 elicited significant production of sIgA responses, and the response was found independently of the route of vaccination. This indicates that *Salmonella* based live vaccines efficiently colonize and replicate in lymphoid organs, and subsequently elicit potent immune responses. Our results are in agreement to the earlier reports, which show that live *Salmonella* based vaccines can efficiently colonize the lymphoid organs and Peyer's patches at high concentrations, and stimulate both mucosal and systemic immune responses [10]. Although sIgA responses are important in limiting viral infections to the host cells, systemic IgG immune responses also contributes to antiviral immunity. In the present study, we found that JOL1863 induced significant production of systemic IgG responses as compared to unimmunized control group. This supports the conclusion that *Salmonella* based vaccines can enhance systemic as well as mucosal immune responses, irrespectively of the route of vaccination.

In addition to systemic and mucosal immunity, cell-mediated immunity is required for chickens to completely recover from influenza infections [30]. The cellular immune responses induced by influenza viruses are necessary for viral clearance from the lungs and are, therefore, critical for chickens to recover from influenza virus infections. In the present study, the Salmonella HA based vaccine not only generated efficient mucosal IgA responses but also stimulated a high level of AIV-specific cellular response. Our results demonstrate that restimulation of PBMCs with HA resulted in significantly higher proliferative responses as compared to the unimmunized control group. This can be explained, at least in part, by a *Salmonella*-dependent improvement in the capacity of DCs to process and present MHC class-I restricted antigens. Further, we evaluated the recall cytokine responses in PBMCS. The nature of the cytokines released after vaccination or *in vitro* restimulation of cells is an important parameter to define the type of the immune response stimulated. Our data demonstrate that there is an increment in Th1 cytokine (IFN-γ) expression by PBMCs in the presence of HA. IFN-γ is an important Th1 cytokine and is critical in the development of cellular immune responses, especially cytotoxic CD8+ responses, which are effective in clearance of viral infections [31, 32]. Further, JOL1863 immunized chickens showed an increment in IL-17, a proinflammatory cytokine, which plays protective roles in host defense against certain pathogens at epithelial and mucosal barriers [33]. A study by Wang et al. shows that IL-17 deficient mice exhibit markedly increased weight loss, more pronounced lung immunopathology, and significantly reduced survival rates upon challenge with the H5N1 infection [34]. Studies have further demonstrated that IL-17 can protect mice against lethal infection with both A/Puerto Rico/8/34 (H1N1) and A/Alaska/6/77 (H3N2) influenza virus [35]. This might explain why JOL1863 vaccinated chickens showed significant protection against the lethal H7N1 influenza virus challenge. We also observed an increment in IL-10 cytokine, which has a clear role in IgA production and generation of potent antibody responses through class switching to IgG1 and IgG3 [36, 37]. These findings clearly indicate that *Salmonella*-based HA vaccine has the potential to stimulate efficient protective immune responses and can protect from pathogenic influenza infections.

Taken together, our strategy of employing the codon-optimized HA amino acid sequence delivered by an auxotrophic mutant of *S*. Typhimurium has proven to be effective in eliciting significant protective immunity in chickens, and induced efficient humoral and CMI responses, irrespectively of the route of vaccination. Thus, *Salmonella* based vaccines constitute a promising technology for the development of more efficient protein based vaccines. Influenza viruses have the potential to continuously evolve to divergent strains, so a consensus HA based vaccine strategy is unlikely to provide a one-for-all solution that is universally desired. Nevertheless, *S*. Typhimurium based HA vaccine strategy can be modified readily and rapidly to adjust to changes in the circulating viral strains. Other studies have demonstrated that *Salmonella* based HA vaccines can protect the mice against H5N1 and H9N2 infections [18, 21]. However, the vaccine strategy described herein warrants further evaluation as a potential prophylactic approach in combating pathogenic AIV to avoid the emergence of HPIV affecting human population. Moreover, future studies are needed to determine the protective efficacy of JOL1863 vaccination against various heterologous H7 subtype virus challenges.

## MATERIALS AND METHODS

### Virus and cell line

The LPAI H7N1 influenza virus was cultivated in the allantoic cavity of SPF embryonated eggs, titered in Madin Darby Canine Kidney (MDCK) cells, and expressed as 50% tissue culture infective dose (TCID_50_). The 50% egg infective dose (EID_50_) of H7N1 was determined in SPF embryonated eggs by Reed and Muench method [38], before use in challenge experiment.

### Construction of an attenuated auxotrophic *Salmonella* Typhimurium mutant expressing consensus HA gene sequence

All full length HA sequences from H7N3, H7N7, and H7N9 viruses available from in early 2012 to June 2015 were downloaded from the NCBI database (http://www.ncbi.nlm.nih.gov/genomes/FLU), and aligned by the Clustal W algorithm from the BioEdit program (version 7.0.9:Tom Hall, Ibis Biosciences, Carlsbad, CA) [39]. The most conserved amino acid at each position was chosen to create a consensus HA protein and the codons of HA were optimized for efficient expression in *S. Typhimurium*. The optimized consensus sequence was then synthesized (Bionee, Kroea) and built into the pMMP65 plasmid, an *asd*+ constitutive expression vector as described previously [23]. The recombinant plasmid, pMMP65-HA, was subsequently transformed into an attenuated auxotrophic mutant of *S*. Typhimurium, strain JOL912, and the resultant clone was designated as JOL1863. The JOL912 strain was constructed by the deletion of the *lon, cpxR*, and *asd* genes from the wild-type *S*. Typhimurium, JOL401 isolate, as described earlier [23], and used as the delivery vehicle for the HA protein. The bacterial strains used in this study are listed in Table 2. To produce the coating antigen for determination of HA-specific antibodies, the consensus HA gene was cloned into pET28a(+) expression vector (Novagen, San Diego, USA) and transformed into *E. coli* BL21 plys strain (Novagen, USA) for protein expression. The expressed protein both in *S*. Typhimurium and *E. coli* was confirmed by Western blot analysis using anti-Influenza A antibody (catalog #, MBS1488599).

**Table 2:** Bacterial strains and plasmids used in this study

Strains/plasmids	Description	References
**Strains**X232	*E. coli* Δ*asd* strain, used for cloning of genes into *asd*^+^ plasmid	Lab stock
JOL401	*Salmonella* Typhimurium wild type, SPI-1 *invAE*^+^*hilA*^+^*avr*^+^; SPI-2, amino acid permease^+^; SPI-3, *mgtC*^+^; SPI4, ABC transporter; SPI5, *pipB*^+^; antigen preparation	Lab stock
JOL912	*Δlon, ΔcpxR, Δasd* mutant *of S*. Typhimurium	[23]
JOL1863	JOL912 with pMMP65-HA1 plasmid	This study
JOL1760	*E. coli* BL21(DE3) pLysS expressing HA via pET28a (+) system	This study
**Plasmids**		
pET28(+)	IPTG-inducible, T7 expression vector, C-terminal 6x His tag, Kan^R^	Novagen, USA
pMMP65	asd+, pBR*ori*, lactamase signal sequence based periplasmic secretion plasmid, 6 His tag, high copy number plasmid	[23]
pMMP65-HA1	pMMP65 harboring consensus HA1 gene	This study

### MoDCs culture and stimulation

PBMCs were isolated from chickens under sterile conditions as described previously [40] and cultured in six-well plates (SPL life sciences, Korea) in complete RPMI-1640 medium containing 10% chicken serum (Sigma-Aldrich, USA), 100 IU/ml penicillin and 1 μg/ml streptomycin. The cells were provided with 50 ng/ml recombinant chicken GM-CSF (Kingfisher Biotech Inc, Saint Paul, MN, USA) and 25 ng/ml recombinant chicken IL-4 (Kingfisher Biotech) for 7 days at 41°C with 5% CO2. The culture media was replaced after every 2 days supplemented with the same concentrations of the cytokines. MoDCs were harvested after day 7 for further experiments. For stimulation experiments, approximately 1 × 10^6^/ml MoDCs were cultured in 6 well plates in complete RPMI-1640 media and treated with either JOL1863 (10 particles/cell) or inactivated H7N1 virus (500 ng/ml) or left unstimulated for 48 hrs. For analysis of DC maturation markers, cells were incubated on ice with FITC conjugated anti-CD11c (Serotec, USA), mouse anti-chicken CD80 and CD40 APC-conjugated streptavidin antibodies (Southern Biotech, USA) as per the manufacturer's instructions. A Miltenyi Biotec flow cytometry (Miltenyi Biotec, Germany) was used to collect 25,000 events after creating an appropriate live gate. FACS data were analyzed using MACSQuantify software (Miltenyi Biotec, Germany).

### Immunization and challenge of birds

All animal experimentation work was approved by the Chonbuk National University Animal Ethics Committee (CBNU2015-00085) and was carried out according to the guidelines of the Korean Council on Animal Care and Korean Animal Protection Law, 2007; Article 13 (Experiments with Animals). One-day-old forty female layer chickens (Corporation of Join hatchery, Republic of Korea) were maintained under standard conditions and provided antibiotic-free food and water ad-libitum. Five weeks later, the chickens were divided randomly into four groups (*n* = 10). Groups 1, 2 and 3 were immunized with 10^9^ CFU of JOL1863, delivered orally (P.O), intranasally (I.N) and intramuscularly (I.M), respectively, while group 4 received 200 μl PBS intramuscularly. Serum and intestinal wash samples were collected on the day of immunization (pre-immunization) and weekly thereafter to assess the HA specific immune responses. Four weeks post-immunization, all the chickens were challenged intranasally with 10^6^ EID50 of H7N1 heterologous virus. The cloacal swabs were taken on 1, 3, 5, and 7^th^ day post-challenge to check the viral copy numbers by RT-PCR assay as described previously [41].

### Hemagglutinin inhibition assay

Hemagglutination inhibition (HI) assay was performed to assess the neutralizing antibodies in the sera of immunized and control group chickens as described previously [42].

### Systemic IgG and mucosal IgA specific HA1 antibody responses

An indirect ELISA was used to measure systemic HA specific IgG antibody levels in the sera and mucosal IgA levels in the intestinal lavage samples, respectively, as described previously [43]. The intestinal lavage samples were collected to determine the secretory IgA (SIgA) concentrations as per the protocol described elsewhere [44].

### Lymphocyte proliferation assay

The lymphocyte proliferation test was performed to evaluate cell-mediated immunity in the immunized groups. Three weeks post-immunization, PBMCs were separated from the blood of five randomly selected chickens per group as described previously [40]. *In vitro* proliferation of PBMCs to HA recall antigen was measured by MTT [3-(4,5-Dimethylthiazol-2-yl)-2,5- diphenyltetrazolium bromide] based assay as described in Dar et al. [45].

### qRT-PCR assay

PBMCs stimulated with either inactivated H7N1 antigen or recombinant HA or inactivated JOL1863 were harvested after 48 hrs, and total RNA was isolated by RNeasy Mini kit (Qiagen, Hilden, Germany) as per the manufacturer's instructions. The cDNA was prepared from equal quantity of RNA (1 μg) using SuperScript™ III Reverse Transcriptase kit (Invitrogen, San Diego, California, USA) as described previously [40] and stored at −20°C until use. Real time PCR assay (qRT-PCR) for gene expression studies was performed with the ABI applied biosystems using Power SYBR Green PCR Master Mix (#4367659, Applied Biosystems, USA) as described previously [40]. The relative amounts of cytokine mRNA present (Supplementary Table 1 normalized with GAPDH) was determined by 2^−ΔΔCT^ method [46].

### Analysis of CD4 T cell population in infected chickens

For analysis of CD4 T cell population after challenge studies, PBMCs were prepared from the whole blood of immunized and control chickens on day 5 post-challenge, and incubated on ice with biotinylated mouse Anti-chicken CD4 (CELL LAB, USA) followed by streptavidin-APC (Southern Biotech, USA) as per the manufacturer's instructions. A Miltenyi Biotec flow cytometry (Miltenyi Biotec, Germany) was used to collect 25,000 events after creating an appropriate live gate. FACS data were analyzed by MACSQuantify software (Miltenyi Biotec, Germany).

### Statistical analysis

Statistical analysis was performed using GraphPad prism 5.00 program (San Diego, CA, USA). Data were analysed by two tailed unpaired student's t-test to compare the data from gene expression studies performed on sorted samples. One way ANOVA with Tukey's multiple comparison test was used between different groups. Data are represented as mean ± standard deviation. p< 0.05 were considered statistically significant.

## SUPPLEMENTARY MATERIALS FIGURES AND TABLES



## Abbreviations

APC: antigen presenting cell
DC: dendritic cell
EID50: egg infective dose 50
HA: hemagglutinin
HIV: Human Immunodeficiency Virus
HPAI: high pathogenic avian influenza
LPAI: low pathogenicity avian influenza
TCID50: tissue culture infective dose.
