# Supplementary material for: A consensus-hemagglutinin-based vaccine delivered by an attenuated Salmonella mutant protects chickens against heterologous H7N1 influenza virus
Source: Oncotarget. 2017 Mar 18;8(24):38780–92. doi: 10.18632/oncotarget.16353 (PMC5503571; doi:10.18632/oncotarget.16353)
Supplement: Supplementary file 1 [file oncotarget-08-38780-s001.pdf]

# A consensus-hemagglutinin-based vaccine delivered by an attenuated *Salmonella* mutant protects chickens against heterologous H7N1 influenza virus

## SUPPLEMENTARY MATERIALS

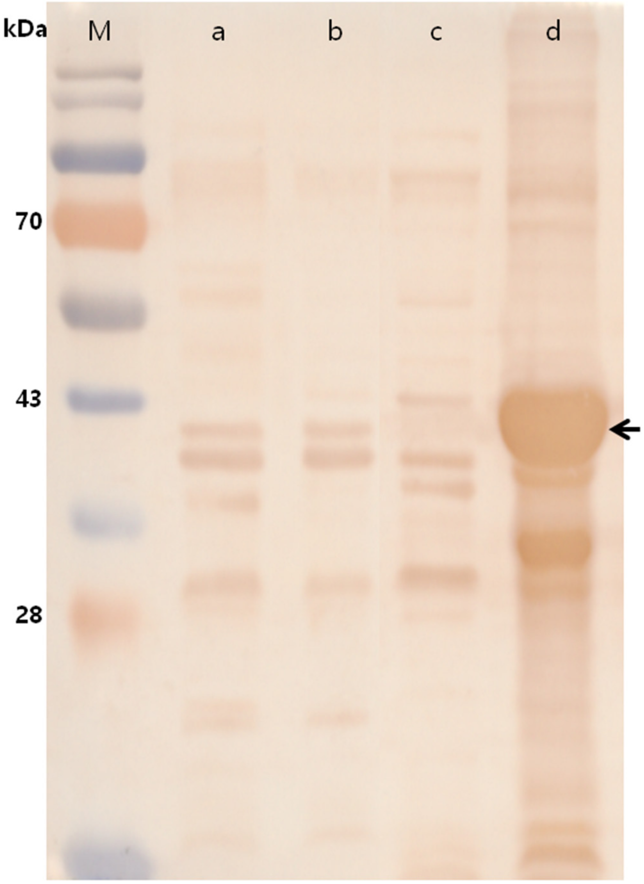


**Supplementary Figure 1: Western blot analysis of HA expressed by JOL1863.** The expression of HA protein expressed by JOL1863 was confirmed by Western blot using polyclonal HA antibody (catalog #, MBS1488599). JOL1863 bacteria harbouring pMMP65-HA plasmid and JOL912 alone (serving as control) were allowed to grow till 0.6 $OD_{600\ nm.}$ Then culture supernatant and bacterial pellet were subjected to Western blot analysis as described in material methods. Lane M, protein Marker; lane a, control supernatant; lane b control bacterial pellet; lane c, supernatant of JOL1863 showing a band of 41 kDa; and lane d, bacterial pellet of JOL1863 showing a 41 kDa band.

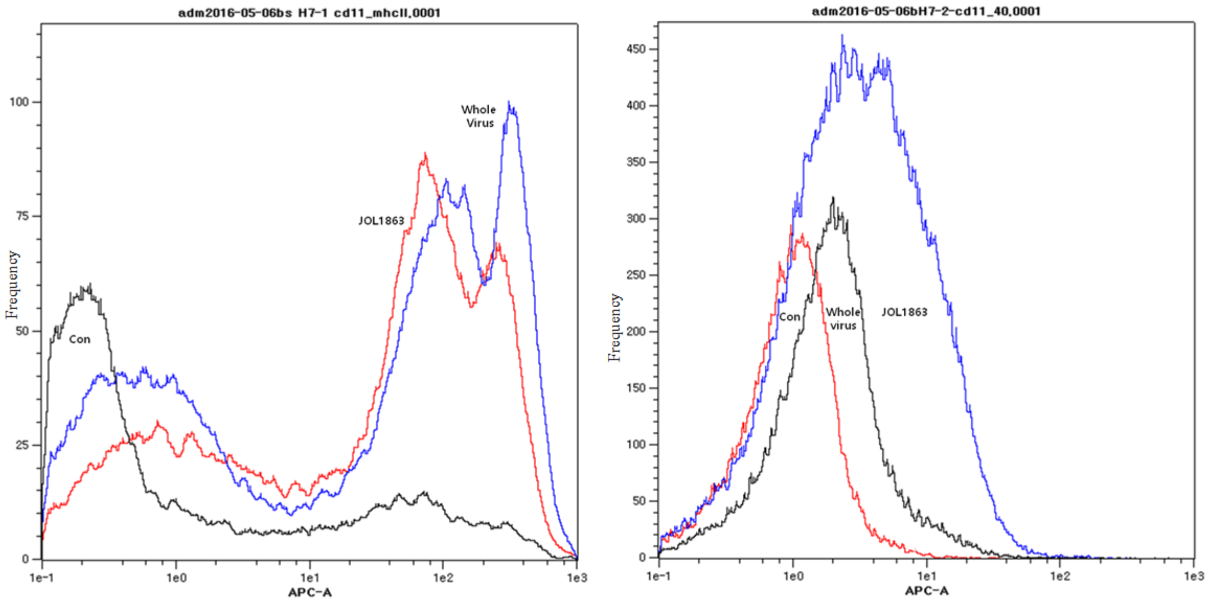


**Supplementary Figure 2: Flow cytometric comparison of chicken immature MoDCs and mature MoDCs with regard to the maturation markers MHC-II and CD40.** Cells were stained with anti-chicken monoclonal antibodies against specific cell surface markers. Immature DCs were stimulated with JOL1863 (10 particles/cell) or H7N1 virus (500 ng/ml) or left unstimulated for 48 hr. The immature DCs (Con) have shown low amounts of MHC II and CD40 while cells stimulated with either JOL1863 or H7N1 virus have shown up-regulation of MHC II and CD40. Data shown are representative of three different experiments.

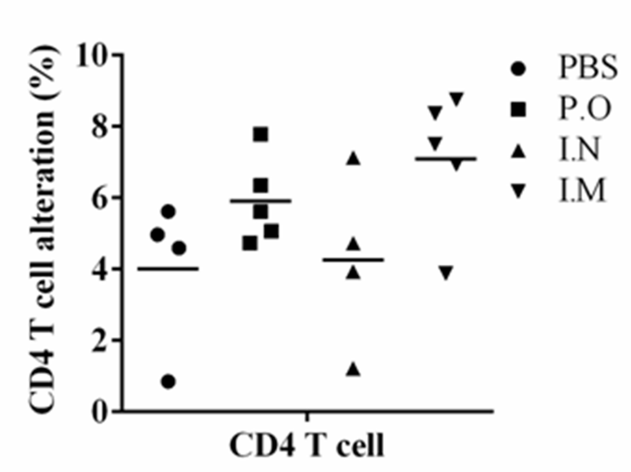


**Supplementary Figure 3: Flow cytometry analysis of CD4 T cell alteration after H7N1 challenge.** After 5 days post challenge, CD4+ T cells in PBMCs were analysed by flow cytometry using anti-chicken CD4 monoclonal antibody. Each data points represent mean+ SD of five chickens per group.

**Supplementary Table 1: List of primer pairs used in this study**

| Primer | Sequence ( 5’ – 3’) | References |
|---|---|---|
| Influenza H7 HA primer F | GCGAAGCTTTTTCGGGATTTCCGGA | Designed |
| Influenza H7 HA primer R | CGCGGATCCGCGGACAAAATCTGCC | Designed |
| Influenza GRAM/7F<br>Influenza GRAM/161R | CTTCTAACCGAGGTCGAAACGTA<br>GGTGACAGGATTGGTCTTGTCTTTA | WHO |
| GAPDH-F<br>GAPDH-R | AGAACATCATCCCAGCGTCC<br>CGGCAGGTCAGGTCAACA | [1] |
| IFN-$\gamma$ F<br>IFN-$\gamma$ R | CAAAGCCGCACATCAAACA<br>TTTCACCTTCTTCACGCCATC | [1] |
| IL10 F<br>IL-10 R | GCTGAGGGTGAAGTTTGAGGAA<br>TGCTGATGACTGGTGCTGGT | [2] |
| IL-17 F<br>IL-17 R | GAGAAGAGTGGTGGGAAAG<br>TCTACAAACTTGTTTATCAGCAT | [3] |

## REFERENCES

1. Jawale C, Somsanith N, Eo S, Park SY, Lee J. Evaluation of Salmonella Gallinarum ghost formulated with MontanideTM ISA 70 VG adjuvant as a vaccine against fowl typhoid. Acta Veterinaria Hungarica. 2015; 63: 401–12. doi: 10.1556/004.2015.038.
2. Liu H, Zhang M, Han H, Yuan J, Li Z. Comparison of the expression of cytokine genes in the bursal tissues of the chickens following challenge with infectious bursal disease viruses of varying virulence. Virology Journal. 2010; 7: 364. doi: 10.1186/1743-422X-7-364.
3. Kim WH, Jeong J, Park AR, Yim D, Kim YH, Kim KD, Chang HH, Lillehoj HS, Lee BH, Min W. Chicken IL-17F: Identification and comparative expression analysis in Eimeria-infected chickens. Developmental and Comparative Immunology. Elsevier Ltd; 2012; 38: 401–9. doi: 10.1016/j.dci.2012.08.002.
